# Potential of Mulberry Leaf Biomass and Its Flavonoids to Improve Production and Health in Ruminants: Mechanistic Insights and Prospects

**DOI:** 10.3390/ani10112076

**Published:** 2020-11-09

**Authors:** Faiz-ul Hassan, Muhammad Adeel Arshad, Mengwei Li, Muhammad Saif-ur Rehman, Juan J. Loor, Jiaxiang Huang

**Affiliations:** 1 Key Laboratory of Buffalo Genetics, Breeding and Reproduction Technology, Ministry of Agriculture and Guangxi Buffalo Research Institute, Chinese Academy of Agricultural Sciences, Nanning 530001, China; f.hassan@uaf.edu.pk (F.H.); lmw1607@163.com (M.L.); 2 Institute of Animal and Dairy Sciences, Faculty of Animal Husbandry, University of Agriculture, Faisalabad 38040, Pakistan; adeel.2203@gmail.com (M.A.A.); shsaifurrehman@yahoo.com (M.S.R.); 3 Department of Animal Sciences, Division of Nutritional Sciences, University of Illinois, Urbana, IL 61801, USA; jloor@illinois.edu

**Keywords:** mulberry leaf biomass, flavonoids, antioxidant, rumen fermentation, methane mitigation, performance

## Abstract

**Simple Summary:**

The economics of livestock production depends upon the feasible feeding resources as feed costs constitute more than 70% of the total expenses of a livestock enterprise. In this regard, searching for alternative and cheaper feeding resources is imperative for economical and sustainable livestock production. Mulberry leaves are an important resource available for feeding livestock, as they possess quite high protein and energy contents as compared to other tree leaves and conventional forages. Moreover, polyphenolic compounds (mainly flavonoids) present in mulberry leaf (ML) possess excellent antioxidant and antimicrobial potential that can beneficially impact animal health and production. Mulberry leaves and its flavonoids have been shown to increase the feed digestibility and milk production in ruminants, while reducing methane emission. Moreover, mulberry flavonoids can positively influence body metabolism and alleviate oxidative stress in animals. This review highlights the importance of this unique feeding resource for ruminants to increase their performance while reducing methane emissions.

**Abstract:**

Leaf biomass from the mulberry plant (genus *Morus* and family *Moraceae*) is considered a potential resource for livestock feeding. Mulberry leaves (MLs) contain high protein (14.0–34.2%) and metabolizable energy (1130–2240 kcal/kg) with high dry matter (DM) digestibility (75–85%) and palatability. Flavonoid contents of MLs confer unique antioxidant properties and can potentially help alleviate oxidative stress in animals during stressful periods, such as neonatal, weaning, and periparturient periods. In addition, mulberry leaf flavonoids (MLFs) possess antimicrobial properties and can effectively decrease the population of ruminal methanogens and protozoa to reduce enteric methane (CH_4_) production. Owing to its rich flavonoid content, feeding MLs increases fiber digestion and utilization leading to enhanced milk production in ruminants. Dietary supplementation with MLFs alters ruminal fermentation kinetics by increasing total volatile fatty acids, propionate, and ammonia concentrations. Furthermore, they can substantially increase the population of specific cellulolytic bacteria in the rumen. Owing to their structural homology with steroid hormones, the MLFs can potentially modulate different metabolic pathways particularly those linked with energy homeostasis. This review aims to highlight the potential of ML and its flavonoids to modulate the ruminal microbiome, fermentation, and metabolic status to enhance productive performance and health in ruminants while reducing CH_4_ emission.

## 1. Introduction

Appropriate and feasible feed resources are required to ensure sustainability of animal production and match ever-increasing global demand for animal products. Crop residues, agro-industrial by-products, shrubs, and tree foliage are usually considered alternate feeding resources for livestock, but their lower digestibility, poor nutritive value (low protein and energy), and unbalanced trace element contents limit more extensive application [1,2]. Inclusion of tropical trees and foliage in cattle ration decreases methane (CH_4_) emission by 10–25%, depending on plant species and level of intake of the ration [3]. Thus, efforts in tropical countries are usually focused on identifying and using local trees and shrubs for livestock feeding owing to their better nutritive value and positive effects on rumen physiology [4,5,6]. Leaf biomass of mulberry trees (family *Moraceae* and genus *Morus*) have been considered traditionally as an alternate livestock feeding resource in China because of their high nutritive profile and flavonoid contents [7]. The mulberry tree is native to the China/Japan area and in the Himalayan foothills but is now cultivated worldwide due to its ability to grow in diverse climates ranging from temperate to tropical areas. Approximately 80 species of genus *Morus* exist around the world; among them, four species viz *M. albus*, *M*. *atropurpurea*, *M*. *multicaulis*, and *M*. *bombycis* are mainly under cultivation [8,9]. Mostly *M*. *albus* and *indica* are utilized in livestock feeding; however, nowadays, paper mulberry (*Broussonetia papyrifera*) is also getting attention because of its antioxidant capacity [10].

Compared to many other traditional forages, mulberry leaves (MLs) contain relatively high protein content (14.0–34.2%), have a high in vivo DM digestibility (75–85%), and are highly palatable due to their succulent nature [11,12]. Protein content of ML is even greater than other traditional forages and is even better than soybean meal, considered a high-quality protein feed for livestock [13,14,15]. Several studies have evaluated the potential use of MLs as a feed ingredient (good quality protein source) in the diets of different livestock species including sheep [16], beef cattle [17], dairy calves [18], and pigs [19]. Fresh leaves are typically fed to animals but ensiled MLs have also shown potential for feeding of beef animals without adversely affecting growth performance and carcass quality [20,21]. The unique nutrient profile, digestibility, and palatability characteristics of MLs make them an excellent protein-rich forage for both monogastric and ruminant animals [22,23].

Owing to an excellent nutrient profile and bioactive components, MLs possess excellent potential as an ingredient in ruminant feed. Despite its nutrient and bioactive rich contents, ML biomass is not extensively utilized for livestock feeding. This review focuses on rich phytochemistry of ML biomass and provides mechanistic insights into the potential of mulberry leaf flavonoids (MLFs) to modulate the ruminal microbiome, fermentation, and antioxidant and metabolic status to enhance productive performance and health in ruminants. The primary objective is to stimulate interest in this natural resource for livestock feeding and encourage researchers to explore the molecular mechanism underlying the biological activities of MLFs.

## 2. Plant Secondary Metabolites of Mulberry Leaf Biomass

Besides their high protein and energy content, MLs also contains a wealth of plant secondary metabolites, especially flavonoids. It is well established that plant secondary metabolites such as tannins, saponins, flavonoids, glucosinolates, mimosine, and essential oils possess different properties including antimicrobial, antioxidant, and anti-inflammatory [24,25]. Among plant secondary metabolites, flavonoids are known as benzo-l-pyrone that have anti-inflammatory, antioxidant, and antimicrobial properties [26,27]. Flavonoids from MLs are famous for their antioxidant potential. Owing to their excellent antioxidant activities, MLFs are of great importance from a biological and pharmacological perspective. Numerous studies have confirmed the antioxidant capacity of MLs or their extracts in rats, cattle, and sheep [18,28,29]. The most promising activities of flavonoids, in addition to being antioxidants, pertain to their potential to modulate different metabolic pathways, especially those linked with energy homeostasis in the body. Due to structural homology with estrogenic hormones, flavonoids exhibit similar anabolic functions through modulation of key lipid and carbohydrate metabolic pathways. Mulberry leaf extract has shown to upregulate the activities of glycolytic enzymes through modulation of gene and transcription factors involved in glucose homeostasis in the liver [29,30,31].

Mulberry leaf flavonoids also exert desirable effects on ruminal function to sustain health and performance [32,33]. Flavonoids alter rumen fermentation dynamics (increased propionate proportion) and favor growth of beneficial microbes like *M. elsdenii* (lactate-utilizing bacteria), which might have desirable effect on animal performance [34]. Mulberry leaf polysaccharides are bioactive components with desirable effects on metabolism and immunity [35].

Supplementation of mulberry leaf powder (MLP) and puerariae flavone in lambs and rams, respectively, improved liver activity by decreasing plasma concentrations of alanine aminotransferase (ALT), aspartate aminotransferase (AST), and lactate dehydrogenase (LD) levels [36,37]. High concentrations of these metabolites in serum are associated with liver and cardiovascular disorders such as Kupffer cell reduction [38,39]. Kupffer cells known as first innate immune cells that have a critical role in maintaining liver functions and protecting the liver from bacterial infections [40]. Some plant secondary metabolites have anti-nutritional factors that can adversely affect health of calves and monogastric animals. However, at an appropriate level, many of them beneficially affect the host metabolism and performance.

## 3. Mulberry Tree Cultivation, Global Distribution, and Leaf Biomass Yield

**Figure 1:**
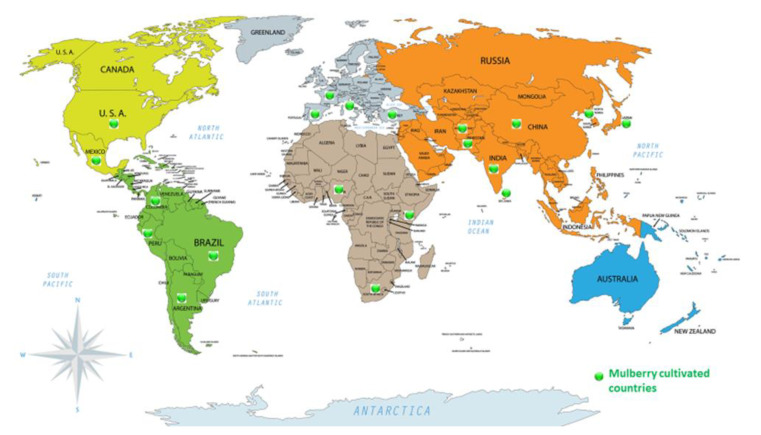
Worldwide distribution of mulberry cultivation.

The mulberry tree has a broader geological distribution (temperate, tropical, subtropical, and arid regions) in different forms (bush, dwarf, and tree). It is being cultivated in different countries of Asia and Europe (from Korea to Spain, France, Italy, Turkey, China, Pakistan, India, Afghanistan, Central Asia, and the Near East); in Africa (North and East Africa, Kenya, and Tanzania); and the Americas (from the United States to Argentina, including Mexico, Central America, Colombia, Peru, and Brazil) (Figure 1). Mulberry can grow in different agro-climatic conditions, such as mountains, plains, irrigated as well as in harsh conditions of humid and semi-arid lands [41,42]. The average yield of leaf and stem biomass as forage ranges from 8 to 52 tons/hectare/year [43,44]. This huge variation in biomass yield is attributed to different mulberry species, agro-climatic conditions, geographical location, soil type, and harvesting method. The most common species of mulberry (*Morus alba* and *indica*) can yield a leaf biomass of approximately 25 to 30 tones/ha/year with a shorter harvesting interval of about 9 to 10 weeks, owing to its ease of propagation and excellent growth characteristics [9]. It makes mulberry a suitable forage for livestock that can be used for feeding fresh or can be processed by making silage to be used for longer periods particularly during the period of fodder shortage.

Most commonly, leaves of the mulberry tree are being used in sericulture for the feeding of silkworm, which is an established industry. Various parts of mulberry (leaf, stem, and root) are utilized in the preparation of various products in pharmaceutical, food, cosmetic, and health care industries [45,46]. From a phytopharmaceutical point of view, the extract of mulberry is utilized as a source of various compound such as carotenoids, coumarins, arylbenzofuran, γ-aminobutyric acid, cyanidin-3-*O*-beta-d glucopyranoside, 1-deoxynojirimycin, ethyl acetate, flavonoids, moran, moranolin, polyphenols, pyrrole alkaloids, polyhydroxy alkaloids, and vitamins [42,47]. Mulberry also has tremendous potential in improving human health owing to its diverse biologically activities including anti-allergic, anti-aging, anti-atherogenic, anti-bacterial, anti-cancer, anti-hypertensive, anti-inflammatory, anti-obesity, anti-oxidant, anti-schistosomal, anti-viral, cardiovascular protectant, free radical scavenging, hepatoprotective, hypoglycemic, lipid-lowering, macrophage activating, neuroprotective, vasoactive, and disinfectant properties [42,48].

## 4. Nutritional Profile of Mulberry Leaves

Alfalfa and berseem are most commonly utilized forages in ruminant diet due to their higher protein (approximately 18–20%) content. Compared with other green leafy vegetables, MLs generally possess greater protein content [49]. Therefore, MLs can be used as an alternate animal feed ingredient to replace plant-based protein sources, due to its high protein content, metabolizable energy (1130–2240 kcal/kg), and digestibility [9,15,50,51,52]. Despite the fact that mulberry is not a legume crop, its leaves still possess a considerable amount of different nutrients, especially protein (14.0–34.2%), carbohydrates (9.7–39.7%), and neutral detergent fiber (19.4–49.7%) on a DM basis (Table 1). The MLs have excellent palatability compared to other fodders (Leucaena and Moringa) as well as high in vitro (>80%) and in vivo (>78%) digestibility in small ruminants [12,53]. Ensiled MLs possess good amounts of crude protein (19.8%), water-soluble carbohydrates (15.6%), and neutral detergent fiber (51.5%), along with trace elements [20].

Notably, MLs also contain different macro minerals (calcium, magnesium, potassium, and phosphorus) and micronutrients such as vitamin C, D, and B1, beta-carotene, iron, and zinc [54,55]. Further, MLs also possess various bioactive compounds (phenolic acids, flavonoids, alkaloids, and γ-aminobutyric acid) with antioxidant and anti-inflammatory function [56,57]. Major antioxidant compounds such as chlorogenic acid, isoquercitrin, and astragalin are also present in MLs [58]. Indeed, MLs also contain a considerable amount of primary fatty acids such as palmitic (26.38% and 25.99%), α-linolenic (34.97% and 37.57%), and linoleic acid (14.76% and 16.05%) [59].

## 5. Anti-Nutritional Factors in Mulberry Leaves

Mulberry leaves contain different antinutritional factors, including oxalates (183 mg/100 g), cyanide (1.01–2.14 mg/kg), tannins (5.32–5.78 mg/kg), and phytate (451–488 mg/kg) on a DM basis [60,76]. Nevertheless, tannins are not considered as an adverse antinutritional factor in ruminants, as they impart some desirable effects such as decreasing protein degradability in rumen coupled with inhibition of methanogenesis and fatty acid biohydrogenation [77,78]. Moreover, MLs also contain iminosugar alkaloids, which can exert an inhibitory effect on mammalian glucosidase enzymes. It is also reported to contain a polyhydroxylated piperidine alkaloid, 1-deoxynojirimycin (0.131–3.483 mg/g), which is a promising competitive inhibitor of intestinal α-glucosidases [79,80,81,82]. Feeding oxalate at 6.75 g/head/d reduced feed intake in goat [83]. Similarly, Rahman et al. [84] showed that feeding oxalate at 30 g/kg DM decreased Ca bioavailability in sheep. Non-ruminants are more sensitive to oxalate than ruminants, as oxalate is degraded by ruminal bacteria. According to Rahman et al. [85], provision of soluble oxalate less than 2% is an appropriate level to avoid oxalate poisoning, although blood Ca level may decrease in ruminants. Provision of potassium cyanide at 3.8 mg/kg/d in goats had an adverse effect on the nervous system [86]. Studies reporting the effect of these antinutritional factors of MLs in ruminants are lacking, so future investigations are required in this regard.

## 6. Structure, Bioavailability, and Absorption of Mulberry Leaf Flavonoids

### 6.1. Structure of Mulberry Leaf Flavonoids

Flavonoids belong to a diverse class of plant compounds that are grouped according to their basic structure: flavonols, flavones, anthocyanins, flavanols, flavanones, and isoflavones [87]. The predominant flavonoids in MLs are flavonols that possess malonyl, acetyl, or other groups as terminal sugars in their basic skeleton [88,89]. Such modifications in the basic skeleton of flavonoids result in the yield of a considerable number of end products with diverse bioactivities. For example, mono- and di-*O*-glycosylated flavonols are the most abundant flavonoids in MLs, which usually include isoquercitrin, astragalin, kaempferol, quercetin 3-(6-acetylglucoside), and rutin with potential anti-inflammatory properties [90,91].

**Figure 2:**
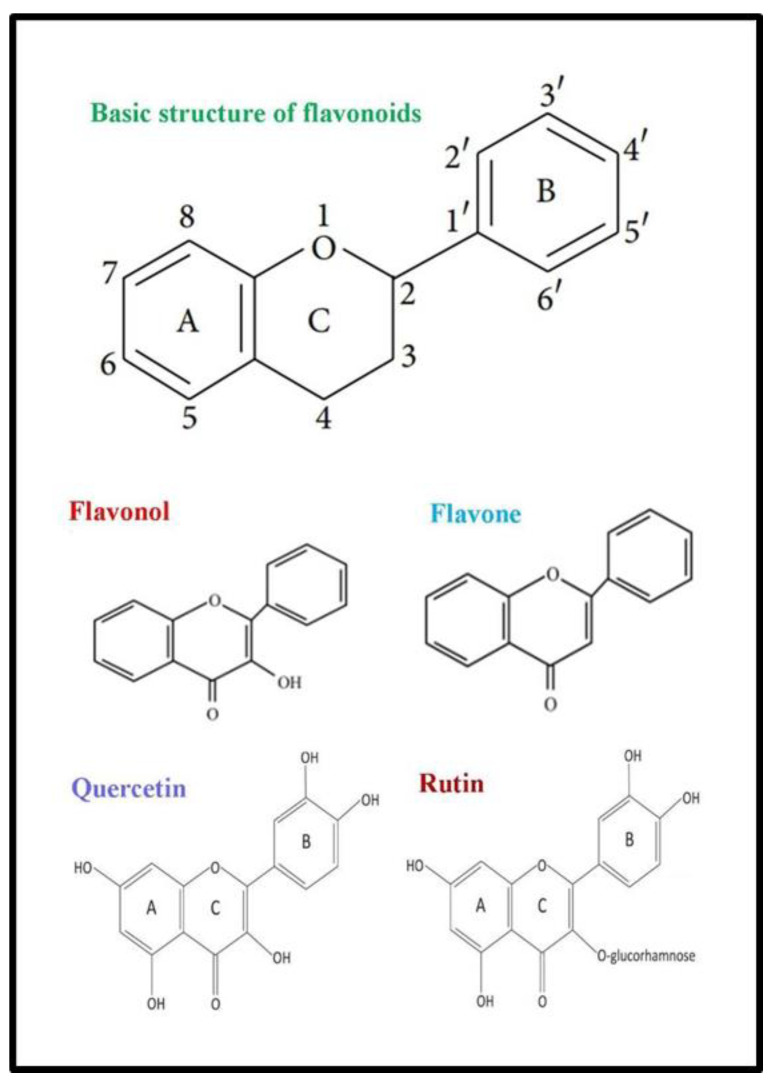
Basic structure of flavonoids present in mulberry leaves.

Flavonoids present in mulberry naturally occur in three forms including aglycones, glycosides, and methylated derivatives (Figure 2). The basic structure of flavonoids is aglycone in which the 6-carbon ring is condensed with benzene to make α-pyrone (flavonols and flavanones) or its dihydro derivatives (flavonols and flavanones). The presence of the benzene ring classifies them as flavonoids (at 2-position) or isoflavonoids (at 3-position). Flavonols differ from flavanones by the hydroxyl group at the 3-position and a C2–C3 double bond [92]. Most flavonoids (except catechin) are present in bound form (glycosides), in which aglycone is attached with a sugar (l-rhamnose, d-glucose, glucorhamnose, galactose, or arabinose) through a *b*-glycosidic bond at position 3 of the C ring [93,94]. Flavonoids in free form (aglycans) can be easily absorbed through the small intestine, but their conjugated forms (flavonoid glycosides) require a first conversion into aglycon before absorption [95]. The presence of a sugar moiety not only determines bioavailability but also reduces its functional properties. Comparison of intraduodenal administration of quercetin in its free aglycone or glucorhamnoside form in Holstein cows revealed greater intestinal bioavailability of free quercetin (aglycone form) as compared to the glycosidic form (glucorhamnoside) [96].

### 6.2. Flavonoid Contents of Mulberry Leaf and Their Bioactivities

The average contents of major flavonoids present in MLs are shown in Table 2. Studies have reported variable contents of total and specific flavonoids in ML, some of which may be attributed to different cultivars of mulberry. Mulberry leaves contain a considerable amount of quercetin (particularly quercetin 3-(6-malonylglucoside), even greater than onions, and is responsible for the potent antioxidant effects of MLs detected under in vitro and in vivo conditions. Furthermore, ML contains other vital flavonoids such as 1-deoxynojirimycin (DNJ), resveratrol, Oxyresveratrol, Cyanidin 3-*O*-β glucoside, and Cyanidin 3-*O*-β rutinoside [97,98].

Plant flavonoids are very diverse in their chemical structures and are ubiquitously present in plants species particularly those used for livestock feeding. Quercetin is the most extensively investigated bioactive flavonoid with important antioxidant, anti-inflammatory, and metabolic potential [101,102]. Quercetin and its derivatives have shown effective hypoglycemic activity through alleviating hepatic oxidative stress [103]. Seven new flavonoids were identified in Korean mulberry (*Morus alba* L.) leaves including kaempferol and quercetin derivatives [88]. Although many studies have characterized flavonoid content, very few reports are available on flavones as compared to flavonols, despite the fact that apigenin and luteolin have also been identified in MLs. Isoflavones possess estrogenic properties and serve as regulators of expression of different genes and transcription factors and antioxidants but also have role in protein tyrosine kinase inhibitors [104]. The largest group of flavonoids is the flavones, which possess diverse biological functions and help plants resist various biotic and abiotic stresses (Table 3). Flavones have shown a positive impact on liver activity by decreasing plasma ALT, AST, and LD levels and also possess antifungal, antiviral, and antibacterial properties [36,105]. Although recent studies have reported flavone content in different mulberry varieties [106], detailed composition of flavonoids in various mulberry cultivars still needs to be investigated further [107].

### 6.3. Ruminal Degradation, Absorption, and Bioavailability of Mulberry Flavonoids

**Figure 3:**
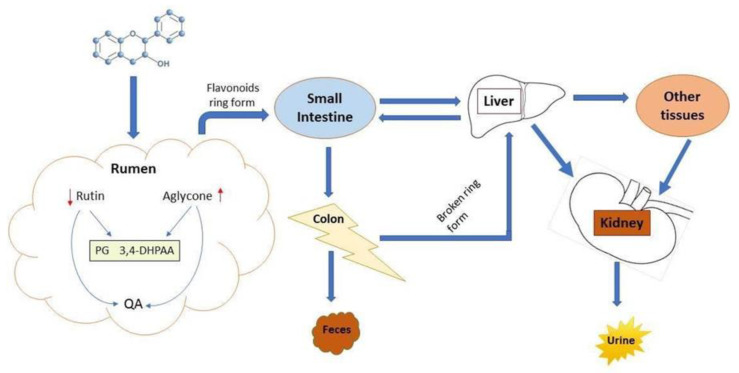
Putative mechanism of flavonoid metabolism and excretion in ruminants.

Generally, flavonoids are absorbed as monomeric form in small intestine of both monogastric species and preruminant calves. However, rumen microbial activity has a positive effect on the utilization of polymeric flavonoids [124]. Removal of the sugar group from aglycone is required to improve bioavailability of flavonoids in the gut. Studies have shown that ruminal microbes can break the *b*-glycosidic bond of rutin (quercetin3-*O*-rutinoside) leading to the release of quercetin and effectively enhancing its bioavailability in the gastrointestinal tract [125]. Furthermore, quercetin along with its methylated (isorhamnetin, tamarixetin) and dehydroxylated (kaempferol) derivatives have been detected in the systemic circulation of non-lactating cows [126], indicating a role of rumen microbes in flavonoid metabolism (Figure 3). Studies on the bioavailability of quercetin in ruminants are limited [96,125]; however, extensive reports are available in monogastric animals [7,127,128]. Intra-ruminal administration of aglycone or rutin revealed a lower absolute bioavailability of quercetin [125]. Nevertheless, similar plasma concentrations of quercetin have been observed after application of the equimolar amount of rutin and free quercetin (aglycone) in monogastric species [127]. Compared with quercetin aglycone, rutin proved to be a much better source of quercetin when administered intraruminally [125]. In contrast, intraduodenal administration of both forms of quercetin resulted in similar concentrations, as observed in monogastric animals [96]. In monogastric species, quercetin bioavailability from rutin is inferior to that of quercetin aglycone [127].

Bioavailability of a compound depends on its fate within the gut. Ruminal microbes can degrade quercetin and its derivatives in vitro, suggesting that quercetin undergoes intensive microbial fermentation in the rumen [129,130,131,132]. Rumen metabolism of quercetin is further demonstrated by the appearance of its degradation products, such as 3,4-dihydroxyphenylacetic acid (3,4-DHPAA); phloroglucinol; and some minor metabolites identified in humans, cows, and pigs [131,133,134]. Oral supplementation of quercetin aglycone and rutin in calves resulted in systemic availability of quercetin and its derivatives tamarixetin, isorhamnetin, and kaempferol in neonatal calves [135]. Additionally, because of underdeveloped rumen in neonatal calves, quercetin aglycone had better bioavailability as compared to rutin [136]. However, in adult ruminants, bioavailability of quercetin aglycone decreases, while rutin increases due to microbial fermentation that results in subsequent partial degradation of quercetin [125,137,138].

Because most of the flavonoids in mulberry are in the conjugated form (rutin or aglycone glycosides), it could be perceived that optimum levels of the active form of flavonoids (quercetin) can be achieved in systemic circulation to impart their subsequent antioxidant and metabolic effects as observed in monogastric species. However, further investigations are warranted to elucidate the extent of degradation of flavonoids in the rumen and their subsequent absorption in the lower gut and systemic bioavailability in different ruminants’ species.

## 7. Feeding of Mulberry Leaves and Its Flavonoids in Ruminants

Mulberry leaves are considered a potential feed for both monogastric and ruminant animals. Some studies have revealed that the inclusion of ML in the ruminant diet can efficiently replace other more expensive protein ingredients [139,140]. Research has been conducted to demonstrate beneficial effects of ML inclusion in neonatal and growing calves, beef cattle, and lactating dairy cattle.

### 7.1. Effects of Mulberry Leaf and Its Flavonoids on Ruminal Development and Calf Health

Nutritional strategies implemented in the pre-weaning period affect the development of the rumen. The intimate cross-talk between the ruminal microbiome, its metabolites, diet, and the host is responsible for successive changes that occur during development of this important organ. For example, volatile fatty acids (VFA) produced by microbes ultimately determine the size and shape of ruminal papilla. These ruminal papillary structures affect microbial colonization, as it provides a niche environment for certain microbes [141]. Thus, for optimization of gut development and sustained microbial colonization, the time of intestinal colonization after birth or during weaning is the most crucial practical consideration in the life of the young animals. Feeding of MLP improved ruminal papillae morphology including width of stratum granulosum and stratum basale. The latter is the primary site for energy metabolism via ketogenesis [71]. Thus, it is likely that the positive effect of feeding MLFs on nutrient digestibility, dietary metabolizable energy, and ruminal fermentation in pre- and post-weaning calves arises from potential direct effects on the epithelium [142,143,144].

Recent studies have shown that feeding MLFs alone and in combination with yeast (*Candida tropicalis*) decreased fecal score during the pre-weaning period of calves [145]. However, during the post-weaning and overall period, fecal scores were similar among all dietary treatments. This reduction in fecal scores happened simultaneously with the increase in concentrations of IgG, IgM, and IgA in response to MLFs and *Candida tropicalis* supplementation. During the pre-weaning period, calves fed flavonoids had greater concentrations of serum epidermal growth factor than those fed yeast. Such findings may be attributed to the fact that the molecular structure of flavonoids is quite similar to steroidal hormones (like estradiol); thus, it can potentially regulate the expression of epidermal growth factor and its receptors [145].

One study has also reported no effect of MLFs on ruminal papillae length, width, and tunica muscularis during the pre-weaning period [18]. Lack of response might have been due to bypassing of flavonoids from the underdeveloped rumen. This idea agrees with the observation that major effects of flavonoids have been observed in abomasum and small intestine. For instance, the thickness of tunica mucosa of abomasum and duodenum was decreased with flavonoid supplementation. As the mucosa plays a major role as a barrier against toxins and bacteria, flavonoid aglycones might bind to mucin via non-covalent interactions and help protect the mucus layer [146]. Thus, the lower mucosal thickness induced by flavonoids could help enhance absorption of nutrients rather toxin. Among systemic effects of MLFs, there are reports of positive impacts on serum growth hormone and insulin-like growth factor-1 (IGF-I) in calves at 56 days of age [144]. Additionally, it has been reported that supplementation of MLFs to *E. coli* challenged calves improved calf health by decreasing fecal scores [147]. Recently, Wang et al. [148] also confirmed that ML promotes goat health by reducing serum leptin concentrations.

### 7.2. Effects of Mulberry Leaf and Its Flavonoids on Animal Health and Performance

Dietary flavonoids not only act as potent antioxidants but also regulate various signaling pathways to guard against oxidative stress at the cellular level [149]. They also possess the ability to enhance absorption and utilization of dietary nutrients, immune response, and lactation performance in animals [150,151]. We demonstrated that supplementation of MLFs increased the concentrations of serum metabolic hormones including estradiol, growth hormone, and prolactin in the lactating buffaloes [152]. Responses might have been attributed to the fact that the molecular structure of flavonoids resembles anabolic steroid hormones (phytoestrogens), which might enhance the regulation of secretion potentially at the level of hypothalamus-pituitary-axis [153]. Albeit with lower affinity, owing, to structural similarities of flavonoids with natural estrogen along with other steroid hormones and their antagonists [154]. They can mediate changes in gene expression similar to estrogens [155]. Mulberry flavonoids particularly quercetin possess many desirable bioactivities and considerable health-promoting effects in animals. Studies in periparturient dairy cows have shown excellent potential of quercetin to alleviate oxidative stress while reducing the extent of liver damage [156]. This effect of quercetin underscores its potential to sustain performance and promote health during metabolically stressful periods such as early lactation [157].

Several studies have been conducted regarding the effects of supplementation of MLs on growth and production performance of animals (Table 4). Supplementing ML pellets up to 600g/d in a rice straw-based diet significantly increased DM intake in beef cattle [158]. Ensiled MLs and sun-dried mulberry fruit pomace have been used in finishing steer diets without impairing productive performance [21]. Dietary supplementation with MLFs at 2 g per head had no adverse effect on feed intake in sheep [7]. Similarly, another study demonstrated that provision of mulberry foliage up to 32% on DM basis in sheep diets resulted in similar DM, organic matter (OM), and NDF intakes [140]. Different tree leaves (*zadirachta indica*, *Melia azedarach*, and *Leucaena leucocephala*) as a forage source for goat has been evaluated by Bakshi and Wadhwa [159] and reported higher DM intake of MLs as compared to others. Liu et al. [16] reported that growing lambs supplemented with different levels of MLs substituting rapeseed meal in ammoniated rice straw diets enhanced feed intake and growth performance. Those results led to the suggestion that MLs could successfully replace rapeseed meal. In the study by Anbarasu et al. [160], a comparison of a leaf meal mixture (*Leucaena leucocephala*, *Morus alba*, and *Tectona grandis*) supplement with groundnut cake or soybean meal was made. Compared with soybean meal, the leaf meal mixture increased daily feed intake and was comparable with the groundnut cake group. However, goats in the leaf meal group had the same average daily weight gain (ADG) as observed with groundnut cake and soybean meal fed goats [160]. The replacement of cottonseed with fresh MLs is possible in growing cattle as the feeding of fresh MLs up to 15% of dietary DM had no adverse effect on DM intake and ADG but improved the feed conversion ratio from 8 to 14% as compared with the control diet [51].

Dietary supplementation with MLFs alone or in combination with *Candida tropicalis* led to greater ADG compared with *Candida tropicalis* alone [145]. Furthermore, combination of *C. tropicalis* and flavonoids exhibited no synergistic effect on calf health compared to flavonoids alone. However, feed intake was similar among all dietary treatments. In another study, dietary supplementation with MLFs improved ADG and feed efficiency of *E. coli* challenged calves without exhibiting any adverse effects on feed intake [18]. Additionally, MLFs in neonatal calves improved feed intake, growth rate, and feed digestibility, especially fat digestibility [117]. Similarly, supplementation of MLFs to *E. coli*-challenged calves improved ADG and feed efficiency [147]. Recently, Ouyang et al. [37] evaluated the feeding to fattening lambs of MLP in high concentrate diets up to 60% in and reported that 15% and 30% level of MLP adequately maintained feed intake and growth performance. However, higher level of MLP (45–60%) had an adverse effect on feed intake and growth performance.

### 7.3. Effects of Mulberry Leaf and Its Flavonoids on Rumen Microbiota and Methanogenesis

There has been an increased public focus on the contribution of enteric CH_4_ emissions from ruminants to global climate change. Enteric CH_4_ is produced by ruminal methanogens through hydrogenotrophic and/or methylotrophic pathways. Nutritional strategies aimed to modulate populations of methanogens and protozoa have proven effective in reducing enteric CH_4_ emissions in ruminants. Flavonoids possess antimicrobial activities [105] and affect ruminal microbial populations, as propolis flavonoids have shown to shift the populations of protozoa and gram-positive bacteria in the rumen [165]. Santas et al. [166] reported that quercetin and kaempferol can inhibit Gram positive bacteria such as *Bacillus cereus*, *Staphylococcus aureus*, *Microcroccus luteus*, and *Listeria monocytogenes*. An in vitro study examined the potential of eight flavonoids (epicatechin, luteolin-7-glucoside, quercetin, isoquercetin, catechin, gallocatechin, epigallocatechin, and epigallocatechin gallate) to mitigate the CH_4_ emission and reported that uteolin-7-glucoside (50 mg/g DM) has promising potential to mitigate CH_4_ and ammonia formation during ruminal fermentation [167]. Another in-vitro study reported that MLFs possess antimicrobial action against *Staphylococcus aureus*, *Bacillus subtilis*, and *Bacillus pumilus* [168]. This is mainly attributed to the ability of flavonoids to interfere with cellular integrity and activity of some Gram negative and Gram positive bacteria as well as protozoa [165,169]. A recent study showed that supplementation of paper mulberry in dairy cows can decrease the relative abundance of genera *Ruminococcaceae UCG-013* and pathogenic bacteria, *Tyzzerella-4* [10]. Owing to their potent antioxidant and antimicrobial activities, MLFs possess an inherent potential to modulate the ruminal microbiome to eventually alter fermentation kinetics and CH_4_ production.

Studies have reported that MLFs can effectively reduce daily CH_4_ output in ewes by reducing the population of protozoa and methanogens, while increasing the abundance of *F. succinogenes*, *R. albus*, and *B. fibrisolvens* [161]. This increase in the population of cellulolytic bacteria was associated with a reduction in protozoal counts. Recently, Olagaray and Bradford [124] reviewed the potential of flavonoids from different plants to reduce CH_4_ emission in ruminants. Inclusion of different flavonoids (flavone, myricetin, naringin, rutin, quercetin, or kaempferol) reduce in-vitro CH_4_ production by 5 to 9 mL/g DM [170]. Dietary supplementation with MLFs (150 mg/kg of diet) improved in vitro DM digestibility, increased total gas production, and VFA, while decreasing CH_4_ production in ruminal fluid of sheep [171].

Mulberry leaves and mulberry fruit also promote abundance of total ruminal bacteria in cattle; however, at the genus level, no effect was observed on *Prevotella Ruminococcus*, *Butyrivibrio*, and *Succiniclasticum* [163]. Likewise, an increased abundance of total ruminal bacteria, including amylolytic, proteolytic, and cellulolytic bacteria were reported with supplementation of ML pellets in beef cattle diet. Among cellulolytic bacteria, more pronounced and promising effects were observed on *R. albus*, which substantially increased with ML supplementation [158]. In addition to direct effects of flavonoids on rumen microbes, their degradation products in the rumen can also effectively modify the microbial metabolism. The breakdown of aglycone ring of flavonoids results in release of phenolic acids, e.g., 3,4-dihydroxyphenylacetic acid from isoquercitrin and quercetin [172,173]. These phenolic end products might take part in the biosynthesis of aromatic amino acids through cinnamic acid pathway. Furthermore, these phenolic compounds such as phenylpropanoic acid and phenylacetic acid have shown to stimulate the growth of cellulolytic bacteria (such as *Ruminococcus albus*) subsequently leading to enhance the cellulose degradation [174,175].

Studies have also suggested that MLFs could also reduce the risk of ruminal acidosis through increasing the populations of lactate-consuming bacteria (*Megasphaera elsdenii*) in the rumen of young animals [124]. Dietary supplementation with MLFs increased the relative abundance of different bacterial phyla, including *Bacteroidetes*, *Proteobacteria*, *TM7*, and *Verrucomicrobi* in *E. coli* K99 challenged pre-weaned calves [147]. At the genus level, greater abundance of *Prevotella*, *Enterococcus*, and *Lactobacillus* was observed in supplemented calves. Owing to the potent antimicrobial activity of MLFs, a significantly lower copy number of *E. coli* K99 was observed in treated calves as compared to controls. The lower copy number of *E. coli K99* in the mulberry group might be attributed to the increased abundance of lactate producing bacteria in the calf gut [147].

Studies have shown that dietary supplementation with MLFs in Holstein calves increased propionate and total VFA concentration [145]. These desirable changes in rumen fermentation may be attributed to the favorable effect of MLFs on dominant cellulolytic bacteria (such as *Ruminococcus albus*) as inclusion of ensiled MLs in the diet of finishing steers led to greater abundance of *R. albus* [162]. Such changes in gut bacteria can potentially increase cellulose degradation leading to better feed utilization and greater VFA yield. Furthermore, a diet containing ensiled MLs and mulberry fruit pomace increased the relative abundance of amylolytic bacteria (particularly *S. bovis* and *Ruminobacter amylophilus*), which influenced starch degradation in the upper gut, while consequently increasing luminal glucose content available for absorption [162]. Both MLs and fruit pomace can produce more fermentable glucose in the gut and also positively influence protein utilization by microorganisms, subsequently leading to better energetic efficiency in ruminants [20,162]. These findings collectively suggest that MLFs can potentially modulate the ruminal microbiome to mediate fermentation kinetics, subsequently leading to better nutrient utilization and performance in ruminants, while reducing the CH_4_ emission.

### 7.4. Effects of Mulberry Leaf and Its Flavonoids on Feed Digestibility and Ruminal Fermentation Parameters

Dietary supplementation with MLP linearly increased the digestibility of organic matter and NDF [71]. Similarly, a linear decrease in NH_3_ and an increase in microbial protein concentrations were observed in sheep (Table 4). Supplementation of MLFs improved apparent digestibility of nitrogen (N_2_) and NDF in ewes, while increasing total VFA. It has been suggested that flavonoids can be used as an alternative source of carbon for the metabolism of the ruminal microbiome, as they are readily degraded in the rumen to yield nonaromatic fermentation products [161]. However, MLFs do not seem to change the ruminal pH and NH_3_ concentration in sheep. In fact, increasing levels of ML pellets in rice straw-based diets increased ruminal NH_3_-N concentrations without affecting pH [158,164]. Similarly, increasing the level of ML pellets in beef cattle increased apparent digestibility of DM, CP, OM, NDF, and ADF. Additionally, MLP also increased the concentration of total VFA, acetate, butyrate, and A/P ratio, except for propionate. Furthermore, greater N_2_ balance was also observed in response to ML pellets in beef cattle [164].

The fact that feeding ML led to similar total DM digestibility in sheep suggested it could be a high-quality forage relative to alfalfa and oat hay [176]. The concentrations of total VFA, acetate, butyrate, and propionate were greater in ML and alfalfa groups as compared with the oat hay group. However, supplementation of MLFs did not change the digestibility of DM, OM, ADF, and NDF compared with control. Furthermore, no effect of MLFs was observed on N_2_ balance and fecal N_2_ excretion [7]. The provision of mulberry foliage at 1.2% of body weight in sheep had no effect on ruminal pH and A:P ratio, but concentration of acetate was greater in the mulberry-fed group. Increased proportion of acetic acid could be due to enhanced digestion of structural carbohydrates, as indicated by greater NDF digestibility [140]. A comparison of different tree leaves indicated that MLs had a relatively high total tract digestibility and N_2_ retention. Studies have suggested that different tree leaves, including MLs supplemented with mineral mixtures and common salt could serve as excellent complete feeds for small ruminants [159]. Compared with rapeseed meal, greater DM degradation of MLs in the rumen of growing lambs have been reported [16].

The DM, OM, ADF, and NDF digestibility and N_2_ balance was similar in goats fed with ML meal and soybean meal [160]. Ruminal pH and NH_3_-nitrogen were unaffected in response to dietary supplementation with MLFs in calves. However, total VFA and propionate concentrations coupled with abundance of propionate-producing bacteria in the rumen increased with MLFs [125]. Feeding of MLs for 60 days enhanced fiber digestion and utilization, subsequently leading to better lactation performance in goat and dairy cattle [177]. They observed a 36.7% increase in milk protein and a 4.5–4.9% increase in lipid content in both species.

### 7.5. Effects of Mulberry Leaf Flavonoids on Oxidative Stress and Antioxidant Parameters

Maintenance of robust antioxidant status is crucial for optimal health and performance particularly in early life. Reactive oxygen species (ROS) are naturally produced during cellular metabolic processes and are neutralized by the antioxidant defense system of the body. Higher production of ROS in stress conditions limit the ability of antioxidant enzymes to maintain balance, leading to oxidative stress [178]. Poor antioxidant response under oxidative stress conditions can adversely affect health and performance of animals [179,180]. To avoid severe consequences of oxidative stress, various nutritional strategies including supplementation of antioxidants and pro-antioxidant compounds have been used in farm animals [181]. Superoxide dismutase (SOD) and glutathione peroxidase (GSH-Px) prevents endothelial and mitochondrial dysfunction by inactivating nitric oxide and inhibits hydrogen peroxide accumulation in the cell, respectively [182,183]; while malondialdehyde (MDA) can affect ion exchange in the cell membrane and can lead to adverse effects, such as changes in ion permeability and enzyme activity [184]. Recently, Hao et al. [10] reported that supplementing paper mulberry increased total antioxidant capacity, SOD, and immune globulin content in dairy cows. Studies have also reported that flavonoids can enhance antioxidant capacity, improve nonspecific immunity, and alleviate oxidative stress by increasing SOD and GSH-Px activity, while decreasing the MDA concentration [185]. The effect is mainly attributed to the ability of flavonoids to act as reducing agents and hydrogen donors to neutralize ROS and remove hydrogen peroxide and superoxide ions [185]. The findings observed in many studies revealed a dual functionality of MLFs to alleviate oxidative stress: (1) by directly interacting with ROS and (2) by increasing the activity of antioxidant enzymes (Table 3).

Different flavonoid compounds possess different antioxidant capacity. For instance, quercetin and morin3-*O*β-d--glucopyranoside have better 2,2′-azino-bis 3-ethylbenzothiazoline-6-sulfonic acid and 2,2-diphenyl-1-picrylhydrazyl (DPPH) free radical scavenging activities [186]. Notably, quercetin-3-*O*-β-d-glucosyl-(1-6)-β-glucopyranose and free quercetin have better DPPH free radical scavenging activity. The MLFs possess better antioxidant activity for scavenging of 2,2-azinobis-3-ethylbenz-thiazoline-6-sulphonate (ABTS) radical, DPPH radical, and total reducing power [168]. Furthermore, Kim and Jang [187] confirmed that rutin, isoquercitrin, quercetin-3-*O*-(6″-*O*-acetyl)-β-d-glucopyranose, and quercetin have the highest superoxide radical scavenging ability and stronger anti-AAPH and Cu^2+^-induced hepatocyte oxidative damage activities. A recent study by Xiaofeng et al. [188] reported comparatively better values of MLs for total antioxidant capacity (60.7 RE mg/g), Fe^2+^ equivalent (42.9 RE mg/g), reducing power (30.5 RE mg/g), DPPH (21.4 RE mg/g), and ABTS (25.1 RE mg/g). They also reported that antioxidant activities of the five above mentioned tests were positively correlated with the content of isoquercitrin, astragalin, and cumulative flavonoids.

Dietary supplementation with MLFs has shown promising effects regarding the improvement of antioxidant capacity and non-specific immunity as well as the reduction in oxidative stress [18]. Various endogenous antioxidant enzymes (SOD and GSH-Px) can convert oxygen-derived free radicals into less toxic forms [189]. Supplementation of MLFs have shown to effectively reduce the oxidative stress by decreasing blood SOD and GSH-Px activities in calves challenged with *E. coli* [18]. Recently, Ouyang et al. [37] observed a significant effect of MLP on different antioxidant enzymes in the liver of fattening lambs. The mRNA levels of Cu/Zn SOD and GSH-Px were increased in a dose-dependent fashion with the supplementation of MLs (15%), revealing the potential ability of flavonoids to activate antioxidant defense system [37]. The MLFs can provide defense against oxidative stress by up-regulating the expression of antioxidant genes mediated by an electrophile responsive element [190]. Silage of ML also has the ability to enhance the antioxidant and immune status of dairy cows [10].

Earlier studies in mice reported that quercetin (a major flavonoid present in ML) can effectively up-regulate the expression of the heat shock proteins (HSP), particularly HSP70 through mediating the ERK/PPARγ signaling pathways [191]. Moreover, the extent of such effects induced by flavonoids on HSP was dependent on the molecular weight (family) of HSPs [191]. Recently, dietary supplementation with MLFs revealed a substantial increase in the expression of serum HSP70 and 90 in lactating buffaloes during the summer season [152]. Furthermore, MLFs significantly reduced the level of a biomarker (MDA) for oxidative stress in buffaloes revealing its potent ability to alleviate oxidative stress. A remarkable decrease in MDA levels up to 75% was observed in buffaloes supplemented with a higher level of MLFs (45 g/d) buffaloes as compared to the control group [152]. These findings support earlier studies regarding a dose-dependent effect of MLFs to mediate ROS [192]. These findings provide strong evidence about the potent ability of MLFs to alleviate oxidative stress caused by different factors, including adverse climate, weaning, physiological state (metabolic transition, early lactation, etc.), and disease.

## 8. Conclusions

The studies reviewed in this manuscript reveal that MLs are a potential resource for livestock feeding owing to their high protein and energy contents coupled with better palatability. During the periods of forage shortage, MLs can serve as a useful feeding resource in ruminants. Flavonoids present in MLs also possess significant antioxidant, antimicrobial, and anti-inflammatory potential. Under the recent scenario of climate change and ban on antibiotics, green feed additives are essentially required to address the heat/oxidative stress, microbial infections, and metabolic disorders in livestock. The leaves of mulberry are rich in protein (14.0–34.2%), minerals (2.42–4.71% Ca, 0.23–0.97% P), and metabolizable energy (1130–2240 kcal/kg) with considerably higher digestibility (75–85%) that make it comparable to high-quality concentrate ration for dairy cattle. With all its rich nutrients and bioactive phytochemicals, ML and its flavonoids possess sufficient potential to address existing and future challenges of livestock feeding. The flavonoid contents in ML biomass confer unique antioxidant properties and can potentially help alleviate oxidative stress in animals under stressful periods such as the neonatal period, weaning, and periparturient period. Most importantly, feeding of ML biomass and its flavonoids can effectively reduce enteric CH_4_ emission from livestock, which is a challenging task under recent climate change scenario.

Despite the excellent potential of MLFs observed in mice and monogastric models, studies regarding the supplementation with MLFs in ruminants are limited. Therefore, further investigation is required to elucidate optimum levels of dietary supplementation with MLFs to ensure proper bioavailability and efficacy in ruminants in terms of promoting health and performance. The potential of MLFs to scavenge ROS and alleviate oxidative stress through enhancing the capacity of endogenous antioxidant defense system needs to be explored at the molecular level to elucidate the mechanism of action of different flavonoids. Furthermore, there is a need to investigate potential effects of MLFs on the endocrine system, different signaling pathways, and their subsequent metabolic effects, particularly regarding the regulation of carbohydrate and lipid metabolic pathways in ruminants.

## Figures and Tables

**Table 1:** Chemical composition of mulberry leaves (%).

Nutrient	Range	Average ^†^	SEM *	References
Dry matter	18 to 30.5	27.3	1.61	[6,21,51,55,60,61,62,63,64,65]
Crude protein	14 to 34.2	21.4	0.88	[6,21,50,51,55,58,60,61,62,63,64,65,66,67,68,69,70,71,72,73,74]
Organic matter	86.4 to 89.8	87.9	0.72	[21,51,61,70]
Fat	3.5 to 8.1	5.1	0.46	[50,51,55,60,61,64,65,67,68,69,70,71,72,73,74]
Fiber	5.4 to 38.4	16.4	2.83	[50,51,55,58,60,61,66,67,68,70]
NFE	25 to 47.9	40.1	5.11	[50,61,67,70]
NDF	19.4 to 49.7	32.6	1.72	[6,21,50,51,55,58,63,64,65,68,69,70,71,72,73,74]
ADF	10.2 to 31.8	40.1	1.46	[6,21,51,58,62,64,65,66,72,73]
Ash	7.6 to 22.4	13.1	0.85	[50,55,60,61,62,63,64,66,67,71,72,73,74]

NFE = nitrogen free extract, NDF = neutral detergent fiber, ADF = acid detergent fiber * SEM = standard error of mean, ^†^ calculated as described by St-Pierre [75].

**Table 2:** Flavonoid and phenolic contents (mg/g) in mulberry leaf biomass.

Total Flavonoids	Rutin	CHA	IQT	QMG	AG	KMG	References
21.36–56.41	0.42–4.31	2.45–10.24	0.70–4.83	0.68–3.05	0.30–1.32	0.46–1.19	[58]
ND	0.90	ND	ND	0.47	ND	0.19	[54]
24.34–58.42	1.09–8.35	4.10–9.67	ND	0.36–13.92	ND	0.07–3.21	[99]
9.84–26.6	ND	ND	ND	ND	ND	ND	[100]
22.5–33.3	2.1	0.13–0.27	3.70–4.01	ND	ND	ND	[59]

CHA = chlorogenic acid, IQT = isoquercitrin, QMG = quercetin-malonylglucoside, AG = astragalin, KMG = kaempferol-malonyl-glucoside, ND = not determined.

**Table 3:** Biological activities of major flavonoids present in mulberry leaf biomass.

Flavonoid	Mechanism	Major Activities	Reference
Quercetin	Inhibition of xanthine oxidase and lipoxygenase, potential ROS scavenger, DPPH scavenging activity, radical oxygen absorption activity	Antioxidant	[108,109,110]
Rutin	DPPH radical scavenging activity, Reducing ROS generation in H_2_O_2_-treated APPswe cells	Inhibition of lipid peroxidation and act as an antioxidant, revert the β-amyloid toxicity	[111,112]
Kaempferol	Improve glucose uptake of 3T3-L1 adipocytes acting as partial agonists of PPARγ, superoxide anion radical scavenging activity	Ameliorate hyperglycemia, antioxidant effects	[113,114]
Isoquercitrin	Lipid-lowering effect and reduced ROS within the Hepatocytes	Reduce oxidative stress	[115,116]
Apigenin	Scavenging ROS and regulation of Fas/FasL pathway	Protects from toxicity and hepatic necrosis	[117,118]
Luteolin	Scavenging reactive oxygen and nitrogen species, inhibiting nuclear factor-kappa B activity and Activator protein 1	Antioxidant and anti-inflammatory activity	[119,120,121]
Astragalin	Suppression of 6-hydroxydopamine-stimulated neurotoxicity, decreased expression of MDA, TNF-α, IL-6, ROS	Alleviation of oxidative stress, cardioprotective Activity	[122,123]

DPPH = 2,2-diphenyl-1-picrylhydrazyl, APPswe = Swedish mutation of amyloid precursor protein, PPARγ = peroxisome proliferator-activated receptor, Fas = cell surface death receptor, FasL = Fas ligand, TNF-α = tumor necrosis factor-α, ROS = reactive oxygen species, MDA = malondialdehyde, IL-6 = interleukin-6.

**Table 4:** Effect of mulberry leaf biomass and its flavonoids on ruminant performance.

Animal	Dose Rate	Major Findings	References
Fattening Hu sheep	Inclusion of MLP at 15, 30, 45, or 60% in concentrate diet	DM intake and average daily gain was optimized up to 30% MLP	[37]
Calves	MLFs at 2 and 4 g/d during pre and poet-weaning respectively	Improved growth performance and feed digestibility	[144]
Ewes	2 g of MLFs in forage diet (6 weeks)	Reduction in CH_4_ emission by 12%	[161]
Simmental crossbred steers	Ensiled MLs (16 weeks)	Higher abundance of *Ruminococcus albus* and *Ruminococcus albus* in the fecal sample	[162]
Simmental crossbred steers	Corn grain and cottonseed meal diet replaced by 8% ensiled MLs group, and 6.3% sun-dried mulberry fruit pomace (16 weeks)	The concentration of total VFA improved with ensiled MLs compared to sun-dried mulberry fruit pomace	[21]
Simmental crossbred steers	Corn grain and cottonseed meal diet replaced by 8% ensiled MLs group, and 6.3% sun-dried mulberry fruit pomace (16 weeks)	Bacterial community composition was similar among the three groups	[163]
Beef cattle	Mulberry leaf pellet supplementation at 200, 400, and 600 g/d with rice straw (21 d)	Improved DM intake, ruminal NH_3_-N, and cellulolytic bacteria	[158]
Beef cattle	Mulberry leaf pellet supplementation at 200, 400, and 600 g/d with rice straw (21 d)	Improved apparent metabolizable energy of DM, CP, organic matter, NDF and ADF	[164]
Sheep	Basel diet supplemented with 2 g of MLFs	Reduced energy losses of CH_4_ emission	[7]
Sheep	Mulberry foliage 1.2% of body weight	Improved total VFA concentration and digestibility of ADF and NDF	[140]
Goats	Feeding of different tree leaves (*Azadirachta indica*, *Melia azedarach*, *Morus alba*, and Leucaena)	*Morus alba* show higher DM intake and digestibility coefficients	[159]
Growing lambs	Replacement of rapeseed meal with MLs in ammoniated rice straw diet (75 days)	Improved feed intake and growth rate	[16]
Goats	50% replacement of conventional supplements with a mixture of leaf meal of (*Leucaena leucocephala*, *M. alba*, and *Tectona grandis*)	Improved DM intake and comparable nitrogen balance with soybean meal group	[160]
Cattle	Compare different grasses (Bermuda grass, elephant grass, and buffalo grass)	Improved digestibility of DM and OM and ME and NE value of the ML compare to other	[51]
Holstein calves challenged with *E. coli*	5% mulberry flavonoids at 3 g/d (36 days)	Improved feed efficiency and gut beneficial bacterial Count	[147]
Calves	MLFs at 3 g/d during pre- and post-weaning period (21–80 d of age)	The ADG was improved post-weaning and overall period with similar feed Intake	[145]
Calves challenged with *E. coli*	MLFs at 3 g/d	Improved ADG and feed efficiency and reduce oxidative stress	[18]
Buffalo	MLFs at 15, 30, and 45 g/d	Dose-dependent increase in milk yield; while a higher level of MLFs also increased milk fat (%) and protein (%)	[152]
Dairy cows	Paper mulberry silage at 13.5% and 18.0%	Increased milk urea nitrogen and decreased somatic cell count with similar milk yield, DM digestibility	[10]

MLs = mulberry leaves, MLFs = mulberry leaf flavonoids, MLP = mulberry leaf powder, ADG = average daily gain, DM = dry matter, OM = organic matter, ME = metabolizable energy, NE = net energy, ADF = acid detergent fiber, NDF = neutral detergent fiber, VFA = volatile fatty acid, CH_4_ = methane.
